# Exploiting DNA Ligase III addiction of multiple myeloma by flavonoid Rhamnetin

**DOI:** 10.1186/s12967-022-03705-z

**Published:** 2022-10-22

**Authors:** Daniele Caracciolo, Giada Juli, Caterina Riillo, Adriana Coricello, Francesca Vasile, Sara Pollastri, Roberta Rocca, Francesca Scionti, Nicoletta Polerà, Katia Grillone, Mariamena Arbitrio, Nicoletta Staropoli, Basilio Caparello, Domenico Britti, Giovanni Loprete, Giosuè Costa, Maria Teresa Di Martino, Stefano Alcaro, Pierosandro Tagliaferri, Pierfrancesco Tassone

**Affiliations:** 1 Department of Experimental and Clinical Medicine, Magna Graecia University, Catanzaro, Italy; 2 Department of Health Science, Magna Græcia University, Catanzaro, Italy; 3 Net4Science Academic Spin-Off, Magna Græcia University, Campus “Salvatore Venuta”, Catanzaro, Italy; 4 Department of Chemistry, University of Milan, Milan, Italy; 5 Institute of Research and Biomedical Innovation (IRIB), Italian National Council (CNR), Messina, Italy; 6 Institute of Research and Biomedical Innovation (IRIB), Italian National Council (CNR), Catanzaro, Italy; 7 Medical Oncology Unit, AOU Mater Domini, Catanzaro, Italy; 8 Presidio Ospedaliero Giovanni Paolo II Lamezia Terme, Catanzaro, Italy; 9 Sbarro Institute for Cancer Research and Molecular Medicine, Center for Biotechnology, College of Science and Technology, Temple University, Philadelphia, PA USA

**Keywords:** Genomic Instability, DNA Ligase III, LIG3, Flavonoid, Polyphenols, Multiple myeloma, DNA repair, Rhamnetin, Natural compounds

## Abstract

**Background:**

DNA ligases are crucial for DNA repair and cell replication since they catalyze the final steps in which DNA breaks are joined. DNA Ligase III (LIG3) exerts a pivotal role in Alternative-Non-Homologous End Joining Repair (Alt-NHEJ), an error-prone DNA repair pathway often up-regulated in genomically unstable cancer, such as Multiple Myeloma (MM). Based on the three-dimensional (3D) LIG3 structure, we performed a computational screening to identify LIG3-targeting natural compounds as potential candidates to counteract Alt-NHEJ activity in MM.

**Methods:**

Virtual screening was conducted by interrogating the Phenol Explorer database. Validation of binding to LIG3 recombinant protein was performed by Saturation Transfer Difference (STD)—nuclear magnetic resonance (NMR) experiments. Cell viability was analyzed by Cell Titer-Glo assay; apoptosis was evaluated by flow cytometric analysis following Annexin V-7AAD staining. Alt-NHEJ repair modulation was evaluated using plasmid re-joining assay and Cytoscan HD. DNA Damage Response protein levels were analyzed by Western blot of whole and fractionated protein extracts and immunofluorescence analysis. The mitochondrial DNA (mtDNA) copy number was determined by qPCR. In vivo activity was evaluated in NOD-SCID mice subcutaneously engrafted with MM cells.

**Results:**

Here, we provide evidence that a natural flavonoid Rhamnetin (RHM), selected by a computational approach, counteracts LIG3 activity and killed Alt-NHEJ-dependent MM cells. Indeed, Nuclear Magnetic Resonance (NMR) showed binding of RHM to LIG3 protein and functional experiments revealed that RHM interferes with LIG3-driven nuclear and mitochondrial DNA repair, leading to significant anti-MM activity in vitro and in vivo.

**Conclusion:**

Taken together, our findings provide proof of concept that RHM targets LIG3 addiction in MM and may represent therefore a novel promising anti-tumor natural agent to be investigated in an early clinical setting.

**Supplementary Information:**

The online version contains supplementary material available at 10.1186/s12967-022-03705-z.

## Introduction

Cancer is a multi-step process in which different hallmarks are progressively acquired as the result of genomic instability [1]. Error-prone DNA repair represents a major source of genomic instability by triggering a mutator phenotype, which finally leads to malignant transformation and progression [2]. In this scenario, Alternative Non-Homologous End Joining (Alt-NHEJ) [3] has been recently characterized as a driver of cancer genomic instability and, at the same time, as a cancer Achilles’ heel which could be exploited by a synthetic lethality approach [4]. Even if Alt-NHEJ acts as backup pathway when classical-NHEJ (c-NHEJ) or Homologous Recombination (HR) is defective, it cannot restore the original DNA sequence due to the absence of a DNA template, while it catalyzes the joining of unrelated DNA molecules, thus promoting chromosomal translocations [5]. This latter event is mainly due to the high flexibility and distinct DNA binding domain of the DNA Ligase III (LIG3), which could allow simultaneous binding of two DNAs producing intermolecular ligations (“jackknife model”) [6, 7].

All three mammalian DNA ligases (LIG1, LIG3, LIG4) contain a catalytic core, consisting of nucleotidyl transferase (NTase) and OB-fold (OBD) domains, and a DNA binding domain (DBD)[8, 9]. In contrast to the other human DNA ligases, LIG3 is distinguished by the presence of an additional N-terminal zinc finger (ZnF) domain that cooperates with the adjacent DBD domain to nick joining and to stimulate intermolecular ligation of two DNAs, a pivotal function of Alt-NHEJ pathway. Interaction between ZnF and DBD domains is mediated by a positively charged groove (PCG) on DBD, which represent therefore a unique feature of LIG3 [6].

Multiple Myeloma (MM), despite the introduction of novel intervention strategies [10–12], is still a lethal hematologic malignancy strongly characterized by deep genomic instability, which accelerates the development of drug resistance and promotes the therapeutic failure [13, 14]. We previously demonstrated that MM cells are highly addicted to LIG3 and PARP1, two pivotal components of Alt-NHEJ [15, 16]. So far, PARP inhibitors (PARPis) have been the only clinically approved drugs for the targeting of Alt-NHEJ and are indicated for the treatment or maintenance of HR-defective breast, ovarian, and prostate cancer [17, 18]. Although PARPis have been associated with long-term disease control, a very common class effect of hematologic toxicities limits the use of these drugs with the need of frequent dose modifications, treatment interruption, and/or discontinuation [19].

An innovative and original strategy for the targeting of Alt-NHEJ repair could be the inhibition of the final step of DNA end joining, which is catalyzed by LIG3. Previous attempts to target DNA Ligases were based on compounds targeting conserved DBD with the aim to prevent DNA ends recognition and finally block Double Strand Break (DSB) repair [20]. Even if interference with LIG3 functions [21] and anti-proliferative activity [22, 23] have been reported in vitro with some of these compounds, to our knowledge, no selective LIG3 inhibitors are presently available for clinical use.

Considering the three-dimensional (3D) LIG3 structure, we have undergone a wide computational screening of natural compounds to identify specific LIG3 targeting agents as potential candidates for effective Alt-NHEJ inhibition. As a result of this wide search, we focused on polyphenols, a class of compounds that has been associated with several benefic effects on human health [24–29]. Within this class of compounds, we identified Rhamnetin (RHM) as the best candidate for LIG3 binding. We here investigated the RHM as a novel LIG3 targeting natural compound in MM, given its addiction to LIG3, to provide a framework for the clinical development of a safe and effective LIG3 targeting strategy for Alt-NHEJ-dependent malignancies.

## Material and methods

For a more detailed description of the methods used, see Additional file 2: methods.

### Docking and thermodynamics studies

The Schrödinger Suite version 2018 was employed as a computational tool for carrying out all molecular modeling simulations in this study (Schrödinger LLC, New York-USA, 2018).

Starting from the three-dimensional structure of the human LIG3, deposited in the Protein Data Bank (PDB) with the PDB code 3L2P [6], our molecular modeling simulations [30] were carried out. This is the only crystallographic structure of the LIG3 catalytic region, comprising the DBD (DNA binding domain), Ntase (nucleotidyltransferase), and OBD domains (central origin-binding domain) (ΔZnF-LigIIIβ protein) in complex with a 22-mer nicked DNA substrate. The LIG3 structure was optimized by means of the Protein Preparation Wizard tool (Protein Preparation Wizard; Epik, Schrödinger, LLC, New York, NY, 2021) implemented in Maestro, using OLPS_2005 as the force field. Hydrogen atoms were added, missing side chains were built using the Prime module, and side chains protonation states at pH 7.4 were assigned [Schrödinger. Protein Preparation Wizard; Schrödinger, LLC.: New York, NY, USA, 2018., Schrödinger. Prime; Schrödinger, LLC.: New York, NY, USA, 2018.]. The domain containing PCG was selected for further computational studies and it was submitted to 10,000 iterations of fully energy minimization adopting the Polake-Ribiere Conjugated Gradient (PRCG) algorithm and the “all atoms” notation of the OPLS_2005 force field [31], as implemented in the MacroModel suite [Schrödinger Release 2018–1, MacroModel, Schrödinger, LLC, New York, NY, 2018.]. Moreover, the implicit solvation model GB/SA water was adopted with the aim to consider solvent effects and the optimization step was performed up to the derivative convergence criterion of 0.05 kcal Å-1•mol-1.

For the Virtual Screening (VS) studies, a comprehensive database of about 493 polyphenolic derivatives contained in foods, named Phenol Explorer, was considered. In particular, a complete set of polyphenols included in the above-mentioned database (ver. 3.6) was downloaded [https://doi.org/10.1093/database/bap024, https://doi.org/10.1093/database/bas031, J. A. Rothwell, J. Pérez-Jiménez, V. Neveu, A. Medina-Ramon, N. M’Hiri, P. Garcia Lobato, C. Manach, K. Knox, R. Eisner, D. Wishart, A. Scalbert, Database, 2013.; https://doi.org/10.1002/minf.201501040] and prepared considering the ionization states at physiological pH (pH: 7.4) using the LigPrep platform of Maestro [ LigPrep, Schrödinger, LLC, New York, NY, 2018.] Since it is estimated that approximately 50% of drug candidates’ failures in the clinical stage are due to unfavorable ADME properties, pharmacokinetic properties should also be treated as an important filtering step [32]. Thus, in our study, this selection was performed before the screening. ADME properties were predicted for all the polyphenolic derivatives, by means of the QikProp platform (ver. 3.5) [QikProp, Schrödinger LLC, New York, NY (USA), 2018], and only those compounds with the best ADME profile were retained.

The VS was performed applying the SP protocol of the Glide (v. 7.8) software [Glide, Schrödinger, LLC, New York, NY, (2018).] and 10 poses per ligand were generated. A grid box of 27,000 Å3 was built by considering a receptor van der Waals scaling of 1.0 and by centering on the two basic residues (Lys323 and Arg327) in the DBD of LIG3. In fact, the groove containing Lys323 and Arg327 contributes to the interdomain interactions between the ZnF and DBD that allow the binding of one DNA end while the catalytic core binds a second DNA end to promote blunt end joining.

Given the absolute lack of a known compound able to target this portion of LIG3, we opted for an arbitrary cut-off equal to -6.50 kcal/mol to filter the scored compounds according to their G-score value. Thus, we selected 5 compounds and, given their very close structural similarity, only the first best hit was purchased for the following biological tests.

### MM cell lines, primary cells, and reagents

Peripheral blood mononuclear cells (PBMCs) and primary cells from MM patient bone marrow aspirates, following informed consent and University Magna Graecia (Catanzaro, Italy) IRB approval, were isolated using Ficoll–Hypaque density gradient sedimentation as reported previously [33]. MM patients’ cells were separated from bone marrow samples by antibody-mediated selection using anti-CD138 magnetic-activated cell separation microbeads (Miltenyi Biotec). The purity of immunoselected cells was assessed by flow-cytometry analysis using a phycoerythrin-conjugated CD138 monoclonal antibody by standard procedures. CD138 + cells from MM patients pt#1, pt#2 and pt#3 were cultured in RPMI-1640 medium (Gibco®, Life Technologies) supplemented with 20% fetal bovine serum (Lonza Group Ltd.) and 1% penicillin/streptomycin (Gibco®, Life Technologies). MM cell lines (HMCLs). KMS-26 were kindly provided by Dr. Giovanni Tonon (University of San Raffaele Scientific Institute, Milan, Italy). AMO1, NCI-H929, U266 were purchased from DSMZ (Braunschweig, Germany). AMO1 bortezomib-resistant (ABZB) were kindly provided by Dr. Christoph Driessen (Eberhand Karls University, Tübingen, Germany). MM cell lines were cultured in RPMI1640 (Gibco, Life Technologies) supplemented with 10% FBS (Lonza Group). HS-5 human stromal cell line (purchased from ATCC, CRL-11882TM) was cultured in DMEM supplemented with 10% fetal bovine serum and 1% penicillin/streptomycin [34]. Co-culture experiments were performed in 6 well plate at a density of 2,5 × 10 5 cells/ ml in 1:1 HS-5 /MM cells ratio [35, 36].

### In vitro transfection of MM cells

Silencer™ select MYC (clone IDs: s9129) were purchased from Invitrogen™ (Thermo Scientific) and were used at 100 nmol/L final concentration. MM cells were transfected using Neon Transfection System (Invitrogen™) (2 pulse at 1.050 V, 30 ms).

### Cell cycle analysis

Analysis of cell cycle was performed by Propidium Iodide flow cytometry assay (BD Pharmingen), according to manufacturer’s instructions. Flow cytometry analysis was performed by Attune NxT Flow cytometer (Thermo Fisher Scientific).

### DSB repair assay

DSB repair assays was performed as previously described [37, 38]. Briefly, EJ2-GFP plasmid (#44,025, Addgene) were linearized with I-SceI (Thermo Scientific) digestion and transfected into 1 × 10^6^ cells at a ratio of 1 μg per well. 48 h after RHM treatment, the numbers of GFP + cells were determined by flow cytometry (Attune NxT, Thermo Fisher Scientific). For each experiment, FACS analyzed a minimum of 20,000 cells.

## Immunofluorescence

Cells were harvested, centrifuged onto glass slides (Cytospin 4, Thermo Scientific), and fixed in 4% paraformaldehyde in PBS, pH 7.4, for 12 min at 22 °C, followed by three 5-min washes in PBS. Cells were permeabilized (0.1% Triton X-100 in PBS, 15 min), washed in PBS (3 × , 5 min each), and incubated for 1 h at 22 °C with blocking buffer (1.5% BSA in PBS). They were reacted > 12 h at 4 °C with primary antibodies listed in the table, washed in PBS (3 × , 5 min each), and incubated for 1 h at 22 °C in the dark, with appropriate secondary antibodies. Cells were washed 3 × in PBS and mounted under coverslips with Vectashield with DAPI (Vector Laboratories). Images were acquired with an SP2 Leica Zeiss confocal laser-scanning microscope with a 63 × oil objective, as previously described [39].

### Animals and in vivo models of human MM

Male CB-17 severe combined immunodeficient (SCID) mice (6 to 8 weeks old; Harlan Laboratories, Inc., Indianapolis) were housed and monitored in our Animal Research Facility. Experimental procedures and protocols had been approved by the Magna Graecia University IRB and conducted according to protocols approved by the National Directorate of Veterinary Services (Italy). Mice were subcutaneously inoculated with 5 × 10^6^ H929 cells and treatment started when palpable tumors became detectable (100–200 mm^3^). Tumor sizes were measured as described [40, 41], and the investigator was blinded to group allocation.

### Statistical analysis

Each experiment was performed at least three times and values are reported as means ± SD. Comparisons between groups were made with Student’s t-test, while statistical significance of differences among multiple groups was determined by GraphPad software (www.graphpad.com). Graphs were obtained using Graphpad Prism version 6.0. p value of less than 0.05 was accepted as statistically significant. The synergistic index was determined as previously described [42].

## Results

### Virtual screening towards positively charged groove of LIG3 DBD

**Fig. 1:**
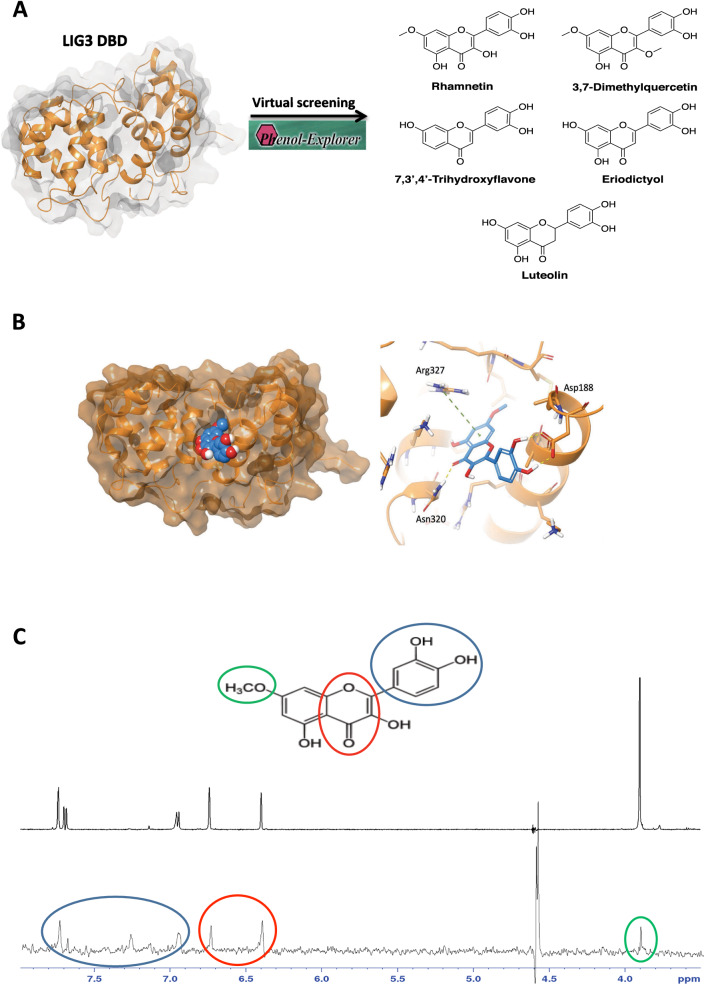
RHM is a LIG3 binding flavonoid. A. Computational screening identify RHM as compound with best docking score to LIG3 positive charged groove on DBD. B. *Left*: Frontal view of RHM (blue spheres) binding mode within the positively charged groove of LIG3 DBD. *Right:* 3D representation of the best pose of RHM (blue sticks) docked into the DBD groove. Hydrogen bonds and π-cation interactions are represented by yellow and green dashed lines, respectively. C. ^1^H (top) and STD-NMR spectrum (bottom) of RHM in presence of LIG3 (100:1). The different groups involved in the binding are highlighted with different colors

LIG3 shows a unique feature on the surface of the DBD, which distinguishes it from LIG1 and LIG4 (Additional file 1: Figure S1A–D). Specifically, a positively charged groove (PCG) is located at the interface of two halves of the DBD between two parallel helices (α1 and α9), adjacent to the DNA binding surface. For these reasons, the domain containing this positively charged groove was selected for further studies of structure-based drug discovery [43, 44]. In the aim to identify a safe Alt-NHEJ targeting strategy, a database containing several polyphenol structures was selected. Indeed, polyphenols are compounds ubiquitously expressed in plants that have been associated with multiple benefits for human health, such as anti-inflammatory, antimicrobial, antiviral, anticancer, and immune-modulatory effects. Thus, the Phenol-Explorer database, the first comprehensive collection of polyphenols contented in foods, was selected for follow-up screening approaches. Initially, the 493 compounds of the Phenol-Explorer database were assessed for Lipinski's Rule of 5 (RO5) and ADMET properties. In fact, the most important aim of drug discovery is the selection of bioactive compounds characterized by drug-likeness properties. Thus, only 152 compounds were able to fulfill the RO5. Afterward, these compounds were screened against the human LIG3 using a structure-based virtual screening (SBVS) approach. Filtering the compounds according to their G-score value, 5 best *hits* were selected (Table 1) (Fig. 1A). Since all 5 *hits* are structurally related, we focused only on the most promising one, which is RHM, a monomethoxy flavone. By analyzing the best docking pose of RHM, we observed that it fitted well within the characteristic groove of LIG3 (Fig. 1B), with the chromone ring facing Lys323 and Arg327, the two most important positively charged residues involved in the interdomain interactions between the Zinc Finger (ZnF) domain and DBD. In particular, the chromone ring established a π-cation interaction with Arg327 and an H-bond with Asn320. Moreover, the binding mode of RHM is further stabilized by the formation of two H-bonds with the side chain of Asp188. Finally, we observed several good hydrophobic contacts, involving Cys183, Ala184, Ala187 and Met335.

**Table 1:** Common name, 2D structure and G-Score value of the best selected hits

Hit	Common Name	2D structure	G-Score *(kcal/mol)*
1	Rhamnetin		-7.14
2	3,7-Dimethylquercetin		-6.89
3	7,3',4'-Trihydroxyflavone		-6.82
4	Luteolin		-6.65
5	Eriodictyol		-6.62

### NMR spectroscopy confirms RHM-LIG3 physical interaction

To validate the findings obtained from the computational analysis, Saturation Transfer Difference (STD) -NMR experiments were performed to confirm the RHM binding to LIG3 recombinant protein.

From the chemical point of view, RHM [2-(3,4-dihydroxyphenyl)-3,5-dihydroxy-7-methoxy-4 h-chromen-4-one, also known as 7-methoxyquercetin] is a member of a class of compounds known as flavonols. These compounds contain a flavone (2-phenyl-1-benzopyran-4-one) backbone carrying a hydroxyl group at the 3-position. RHM is characterized by ^1^H-NMR spectroscopy and the spectrum is reported in Fig. 1C (top). STD-NMR analysis of ligand-target complex was based on the nuclear Overhauser effect that allows the observation of the ligand resonance signals [45, 46]. The term binding epitope is frequently used in the STD-NMR literature to characterize the hydrogens of the ligand that are closer to the protein upon binding and became visible in STD spectra.

STD-NMR analysis of RHM in the presence of LIG3 (Fig. 1C, bottom) showed a specific binding between the ligand and the protein. The resonance of all protons of the molecule have been observed. The resonance of 3,4 dihydroxyphenyl group is shown with blue circles, the two protons of chromenone moiety in red, and the methoxyl group in green. All protons of the molecule are involved in the binding and the quantitative analysis of STD data (short protein–ligand distances produce a strong intensity of the corresponding STD signals) showed that the chromenone ring is the moiety with the strongest interaction (as suggested by docking calculation, this is the part that fits well into the binding pocket). A lower intensity can be detected for the protons of the dihydroxyphenyl ring. The comparison with the docking model suggests that the interaction for this moiety is mediated by the formation of hydrogen bonds with the hydroxyl groups, the presence of D_2_O in the buffer for NMR analysis causes the exchangeable protons to be replaced by ^2^H, which is inefficient for transferring saturation. Hence, ligand protons close to protein exchangeable protons receive less saturation and the contacts mediated by polar groups will show a relative low STD signal. The binding epitope by STD spectrum is comparable to the binding mode suggested by the computational model. Furthermore, the contribution of exchangeable protons (showing very low intensities in the 1D spectrum, Fig. 1C top) by OH groups become visible between 7.1 and 7.3 ppm, indicating that they are engaged in a hydrogen bond with the protein (and therefore they are less prone to exchange with the solvent). Finally, to verify the specificity of the binding, STD experiment was performed in presence of unfolded LIG3 protein. Notably, no RHM binding to LIG3 was observed in this condition (data not shown), suggesting the absence of nonspecific interaction.

Taken together, these data demonstrate that RHM binds directly to LIG3.

### RHM exerts anti-MM activity in vitro

After the demonstration of RHM binding to LIG3, the effect on cell proliferation and survival of this flavonoid was investigated in a model of disease such as MM, which is highly dependent on LIG3-driven DNA repair [6].

**Fig. 2:**
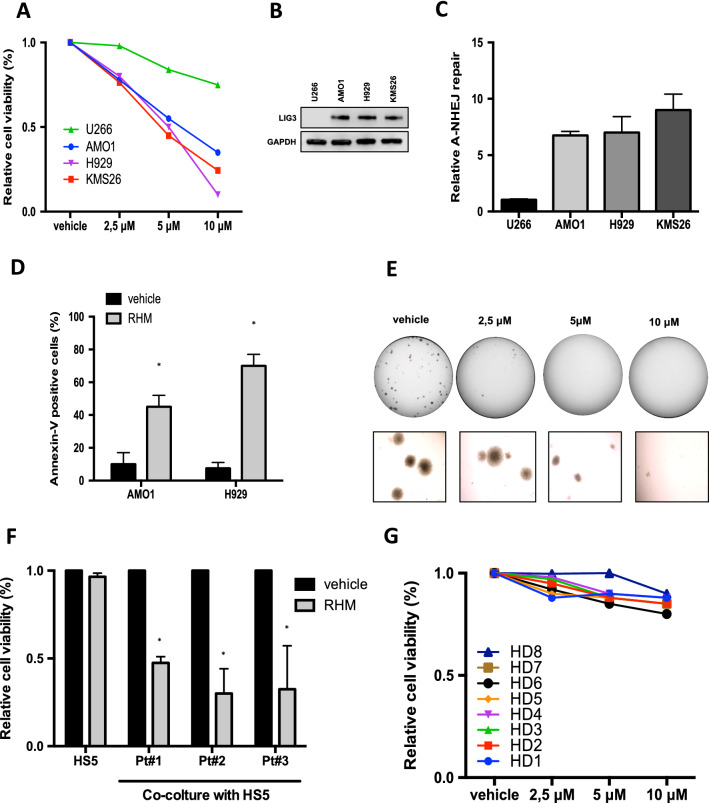
RHM exerts anti-MM in vitro. A. Indicated MM cells were treated with increasing dose of RHM. CTG assay was performed 5 days from treatment. B. Immunoblot of LIG3 performed on plasmacells from MM cell lines. GAPDH was used as a loading control. C. Basal Alt-NHEJ repair of indicated MM cell lines. U266 repair activity was used as internal reference. D. Annexin V assay on AMO1 and H929 cells 4 days from RHM treatment (5 μM). E. Colony formation of H929 cells treated with vehicle or increasing dose of RHM. Light microscopy after 2 weeks is shown. F. Cell viability of CD138 + cells from 3 different MM patients co-cultured with HS-5 stromal cells and treated with RHM 10 μM or vehicle. The assay was performed 5 days after treatment. G. Healthy donors’ PBMCs (n = 7) cells were treated with increasing dose of RHM. CTG assay was performed 5 days from treatment. Results are representative of three experiments, ± SD. *, P < 0.01

In vitro, RHM exerted a significant anti-proliferative activity in 3 out of 4 MM cell lines (Fig. 2A), according to LIG3 expression and Alt-NHEJ activity. Indeed, while the half maximal inhibitory concentration (IC50) of ≈ 5 μM was reported in NCI-H929, AMO1, and KMS26, significant resistance to RHM was observed in U266 cells, which express the lowest LIG3 protein level and Alt-NHEJ repair (Fig. 2B, C). Consistently, by analyzing protein levels of c-NHEJ-dependent LIG4 (Additional file 1: Figure S2A), an opposite pattern of expression to LIG3 and an inverse correlation with RHM sensitivity were found, further suggesting that RHM effects on MM cell viability are driven by LIG3-repair dependency. To characterize the induction of cell death, an Annexin V staining was used. RHM induced apoptotic cell death in AMO1 and NCI-H929 cells, (Fig. 2D, Additional file 1: Figure S2B), which was abolished by pan-caspase inhibitor Z-VAD-fmk (Additional file 1: Figure S2C), while no effects were observed on U266 cells (Additional file 1: Figure S2D). Clonogenicity of treated MM cells was also inhibited (Fig. 2E, Additional file 1: Figure S2E), and Haematoxylin and Eosin (H&E) staining showed chromatin fragmentation of treated cells (Additional file 1: Figure S2F). Importantly, RHM impaired the viability of primary MM plasma cells co-cultured with stromal cells (Fig. 2F), thus overcoming the pro-survival role of the MM-microenvironment. Consistently with tumor-selective killing activity, RHM did not affect viability of normal peripheral blood mononuclear cells (PBMCs) from healthy donors (n = 7), thus suggesting a good safety profile (Fig. 2G).

### RHM triggers DNA damage response (DDR) in MM cells by reducing chromatin LIG3 binding

**Fig. 3:**
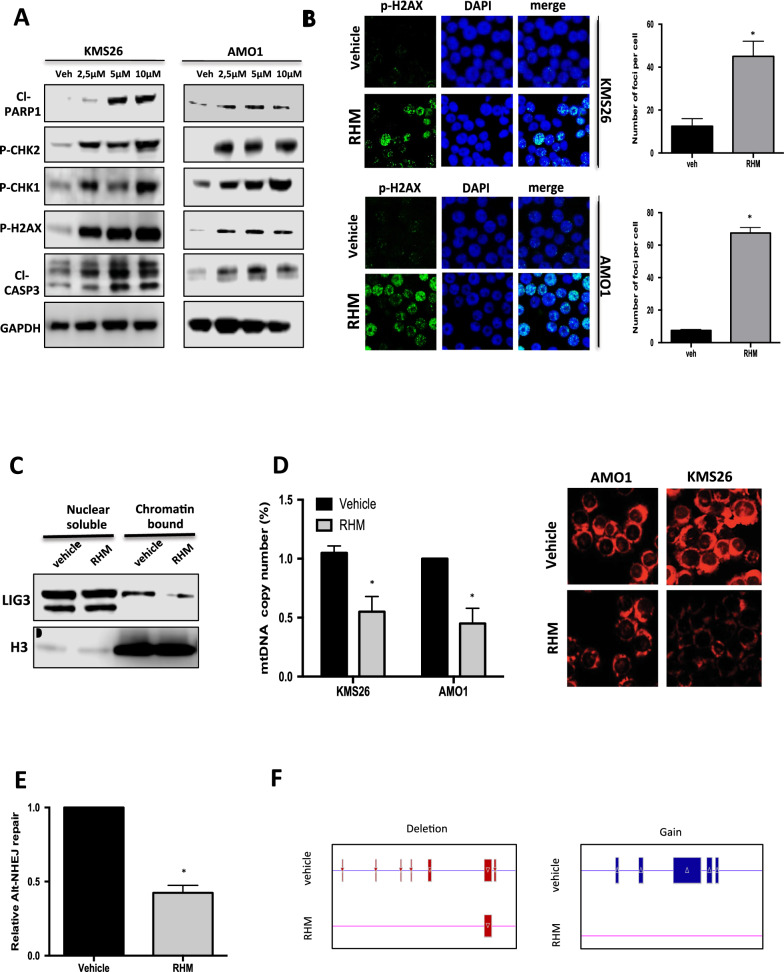
RHM treatment counteracts LIG3 activity and induces DNA damage in MM cells. KMS26 and AMO1 cells were treated with increasing dose of RHM or vehicle. A: Immunoblot analysis of DDR markers was performed 48 h after treatment. B: γ-H2AX foci evaluation by immunofluorescence 48 h after treatment. Representative images of unrepaired DSBs are shown. DAPI (blue) was used for nuclear staining. C**.** Immunoblot analysis of nuclear soluble and chromatin bound fractions prepared from AMO1 cells treated with or without RHM (5 μM). D. KMS26 and AMO1 cells were treated with RHM (5 μM) or vehicle. *Left:* Mitochondrial DNA copy number, as measured by qRT-PCR 48 h after treatment. *Right:* Mitochondria were stained with Mitotracker Red and visualized by fluorescence microscopy 48 h after treatment. E. Alt-NHEJ repair was evaluated by EJ2- GFP assay on AMO1 cells 72 h after treatment with RHM (5 μM) or vehicle. F. Affymetrix CytoScan HD Array analysis, using genomic DNA from AMO1 treated with RHM (2,5 μM) or vehicle. Representative images of deletions or gains acquisition on chromosome 16 (16p11.2) and 1(1p36.3), respectively. Red lines represent deletions, while blue lines represent gains Results are representative of three independent experiments ± SD. *, P < 0.01.

Since LIG3 plays a critical role in DNA DSBs repair, the effect of RHM on DNA damage response was investigated. Importantly, RHM induced a relevant increase in DNA DSBs with a relevant activation of DDR and apoptosis signaling, as demonstrated by increased phosphorylation of CHK1, CHK2, H2AX, occurring together with PARP1 and caspase-3 cleavage (Fig. 3A, B). Cell cycle analysis revealed also G2-arrest following RHM treatment, which was abrogated by caffeine (Additional file 1: Figure S3A), thus suggesting that checkpoint activation induced by RHM treatment depends on DDR signaling [7].

To investigate the molecular mechanisms underpinning the increase of DNA damage, functional analysis was carried out. First, AMO1 cell lysates were fractionated into nuclear-soluble and chromatin-bound fractions after RHM treatment, to evaluate interference on LIG3 nick-sensing activity. Notably, a decrease of LIG3 chromatin binding, as compared to nuclear-soluble fraction was observed (Fig. 3C), suggesting that RHM affects LIG3 recognition of DSBs impairing their repair. Next, the effects on mtDNA metabolism were investigated, given the exclusive role exerted by LIG3 in mitochondria DSB repair. Importantly, RHM treatment reduced mtDNA content (Fig. 3D *left*), as result of un-repaired mtDNA damage. Consistently with mtDNA function impairment by RHM, a reduction of mitochondrial mass and an increase of reactive oxygen species production (Fig. 3D *right*, Additional file 1: Figure S3B) were also observed. Finally, a validated plasmid-rejoining assay as a measure of Alt-NHEJ repair was performed. Notably, MM cells treated with RHM showed joining reduction as compared to the vehicle alone (Fig. 3E). Consistently with down-modulation of Alt-NHEJ repair pathway, SNP-array analysis confirmed an overall reduction of copy-number variations (CNVs) acquisition in RHM-treated cells (Fig. 3F), Additional file 1: Figure S3C), further indicating that RHM impairs LIG3-driven error DNA repair.

### In vivo activity of RHM in MM

**Fig. 4:**
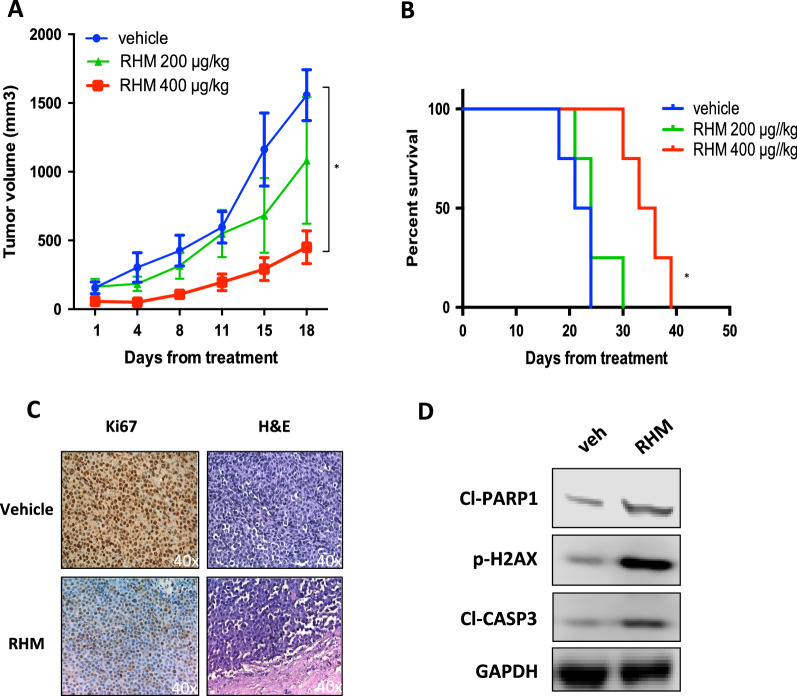
RHM exerts anti-MM activity in vivo. A. In vivo growth of NCI-H929 xenografts treated with RHM (200 μg/kg and 400 μg/kg) or vehicle. Treatment was administered daily ip. *Left panel*: averaged tumor volume of each group ± SD is shown. *p < 0.05. *Right panel*: survival curves (Kaplan–Meier) of each group (log- rank test, *p < 0.05). Survival was evaluated from the first day of treatment until death or sacrifice. Percentage of mice alive is shown. C. *Left panel*: IHC analysis (40x) from a representative H929 xenograft per group (vehicle and RHM 400 μg/kg) for Ki67 and H&E expression. *Right panel*: Immunoblot of p-H2AX, cleaved-caspase-3, cleaved-PARP1 in lysates from a representative NCI-H929 xenograft per group (vehicle and RHM 400 μg/kg). GAPDH was used as a loading control

To investigate the translational relevance of our in vitro findings, in vivo anti-MM activity of RHM was evaluated in NOD-SCID mice bearing sub-cutaneous NCI-H929 xenografts. RHM intraperitoneal (i.p.) daily treatment (400 µg\Kg\day) resulted in a significant tumor-growth inhibition (Fig. 4A), which translated into survival prolongation of treated mice (Fig. 4B). Moreover, Immunohistochemistry (IHC) analysis showed decreased Ki67 expression, tumor regression, and necrotic pattern by H&E staining, thus confirming in vivo antitumor activity of RHM (Fig. 4C)*.* Consistent with in vitro data, an increased expression of DDR and apoptosis markers in tumors retrieved from RHM-treated animals were found, such as phosphorylation of H2AX and Casp-3 and PARP1 cleavage (Fig. 4D).

### Bortezomib resistant cells are highly sensitive to RHM

**Fig. 5:**
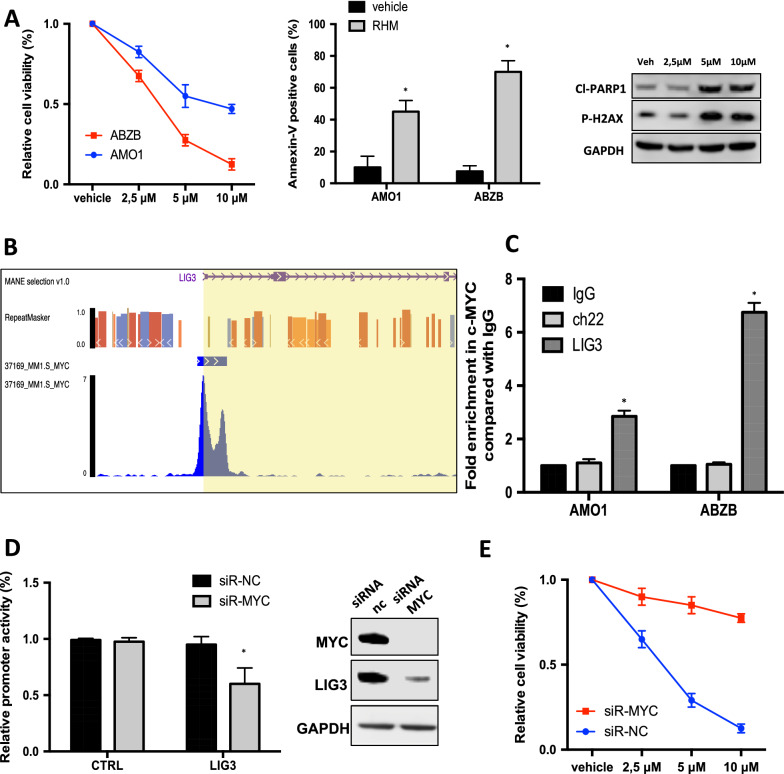
Bortezomib-resistant cells are highly sensitive to RHM. A. *Left panel:* Cell viability of AMO1 and ABZB, treated with vehicle or increasing dose of RHM for 5 days. *Middle panel:* Annexin V assay of AMO1 and ABZB, treated with vehicle or RHM (5 μM) for 48 h. *Right panel:* Immunoblot of cl-PARP1, and p-H2AX performed on AMO1 and ABZB 48 h from treatment. GAPDH was used as a loading control. B. Graphical results of bio-informatic screening (cistrome.org) showing MYC binding to LIG3 promoter. C. qPCR for ch22 and LIG3 promoter performed after Chip with MYC antibody in AMO1 and ABZB and IgG controls. D. *Left panel*: Promoter activity of transfected LIG3 and negative CTRL promoter constructs in ABZB cells co-transfected with either siRNA-NC or MYC-siRNA. *Right panel*: ABZB cells were transfected with siRNA-NC or MYC-siRNA. Immunoblot of LIG3 and MYC was performed 48 h after transfection. E. Cell viability of ABZB transfected with siRNA-NC or siRNA-MYC and treated with vehicle or increasing dose of RHM for 5 days

Hyper-activation of the Alt-NHEJ pathway was been associated with drug resistance in several malignancies [23, 47], including in MM wherein higher LIG3 and PARP1 expression and Alt-NHEJ activity are associated with Bortezomib resistance [15, 16]. On these premises, the activity of RHM on the survival of Bortezomib-resistant cells (Additional file 1: Figure S4A) was then investigated. Notably, RHM exerted higher anti-proliferative activity in AMO1 Bortezomib-resistant (ABZB) cells as compared to their isogenic counterpart AMO1 (Fig. 5A).

To investigate the molecular mechanisms underlying the higher ABZB-sensitivity to RHM and considering the crucial role exerted by MYC in the induction of PARP-mediated Alt-NHEJ [16, 48], a potential role of MYC on LIG3 expression was investigated. Notably, analysis of MM patients’ dataset (GSE24080), disclosed a significant positive correlation between MYC and LIG3 mRNA expression (Additional file 1: Figure S4B). Consistently, a bio-informatic screening (cistrome.org) showed significant enrichment of MYC binding consensus sequences in the LIG3 promoter (Fig. 5B). To wet validate the *in-silico* data, MYC binding to the LIG3 promoter was first confirmed in AMO1 and ABZB by Chip analysis (Fig. 5C), according to MYC expression (Additional file 1: Figure S4C). Next, we demonstrated that MYC knockdown led to down-regulation of LIG3 protein expression and promoter activity and led to impaired RHM sensitivity (Fig. 5D, E), further suggesting MYC as a master regulator of LIG3-driven genomic instability and as a potential predictor of response to Alt-NHEJ inhibitors.

## Discussion

Deregulation of DNA repair is a hallmark of cancer and relies on error-prone mechanisms, such as Alt-NHEJ, which finally increase the mutation rate and burden [49]. Indeed, cancer cells are more dependent upon low-fidelity pathways for DSB repair as compared to normal cells, thus pointing to Alt-NHEJ as potential cancer Achilles’ heel [50–52]. Although PARPi, the only Alt-NHEJ inhibitors presently approved in the clinical setting, have demonstrated therapeutic activity in a variety of randomized phase II and III trials, pieces of evidence have caused concerns about the increased incidence of adverse events, such as myelodysplastic syndrome and acute myeloid leukemia [24, 53]. For these reasons, the empowerment of alternative ways to target Alt-NHEJ repair is eagerly awaited. In this context, inhibition of POLQ, a relevant regulator of Alt-NHEJ machinery, has recently been shown to be effective in HR-deficient tumors [54].

On these premises, we investigated a novel strategy to selectively target LIG3, a pivotal component of Alt-NHEJ. We focused on the 3D structure of LIG3 characterized by the presence of a positively charged groove on DBD, which is crucial for its interaction with N-terminal ZnF domain [6]. Importantly, this unique LIG3-ZnF/DBD binding module is essential for nick-sensing and intermolecular DNA joining activity which produces the genomic rearrangements generated by Alt-NHEJ [55]. This module was selected as the target of inhibitory small molecules of natural origin with a favorable safety profile. We performed a wide computational screening of natural compounds focusing on polyphenols, the most common group of natural bioactive agents in the human diet [24]. Polyphenols are found ubiquitously in plants and are characterized by low toxicity toward normal cells and favorable pharmacokinetic profiles. Several studies support the general healthy value of polyphenols for their well-known protective/preventive effects in a variety of diseases, such as cardiovascular, neurodegenerative, and cancer [56–59]. This is not surprising taking into account that about 70% of cancer drugs are of natural origin or are derivative compounds, and several biologically active agents exert direct anti-tumor activity and/or tone down the side effects of chemotherapeutic drugs, providing benefit to cancer patients [60].

Among several polyphenols investigated, we identified RHM by *in-silico* screen as the best candidate to fit within the 3D groove of LIG3 DBD. RHM is a flavonoid belonging to the flavonol family [61], it derives from quercetin, with the characteristic methylation of the oxygen in position 7 and can be isolated from cloves [62]. After demonstrating by NMR 3D binding of RHM to LIG3, we investigated the effects induced on proliferation and survival of MM cells, a model of highly LIG3-addicted disease, by an orthogonal approach.

We here provide evidence that RHM exerts anti-MM activity in vitro and in vivo, via LIG3 targeting, as demonstrated by correlative and functional analyses. Consistently, the treatment with RHM significantly inhibited nuclear and mtDNA repair, strongly increasing unrepaired DNA damage that finally led to apoptotic cell death of MM cells, and phenocopying LIG3 knockdown [15]. Although RHM has shown pre-clinical anti-tumor activity in different solid tumors [63, 64], this is the first demonstration of its anti-cancer efficacy on hematological disease.

So far, some attempts have been made to target the conserved DNA Binding Domain (DBD) of DNA Ligases, with the aim to prevent DNA ends recognition and finally block DSBs repair [20, 65]. At present, however, to the best of our knowledge, RHM is the first natural compound able to target LIG3, potentially avoiding the detrimental effects deriving from contemporary inhibition of the other DNA ligases in normal cells. Importantly, we demonstrate that RHM is highly effective against bortezomib-resistant cells and antagonizes the onset of new genetic changes in MM cells, thus suggesting a protective role against Alt-NHEJ-driven genomic instability, which triggers disease progression and drug resistance. In this context, although we cannot exclude a direct inhibition on the proteasome, such as described for other flavonoids, we did not observe any synergistic effect or sensitization to bortezomib in MM cells treated with RHM (data not shown). This could be consistent with the evidence that flavonoids could instead inhibit bortezomib-induced apoptosis by chemical reactions with the boronic acid group in the bortezomib structure [66]. Finally, we sought to determine the mechanism leading to higher RHM sensitivity in bortezomib-resistant cells. We demonstrated the activity of MYC as a promoter of LIG3 mRNA expression and, therefore, as a master regulator of Alt-NHEJ in MM. This finding is in line with our data reporting resistance to RHM in U266 cells which are indeed MYC defective [67] and with other previous observations that highlighted a higher mutational load in relapsed refractory MM (RRMM) and a crucial role exerted by MYC expression as a predictive factor to DDR inhibitors response, [68–70], further confirming that Alt-NHEJ function may be more complex than a simple back-up system.

**Fig. 6:**
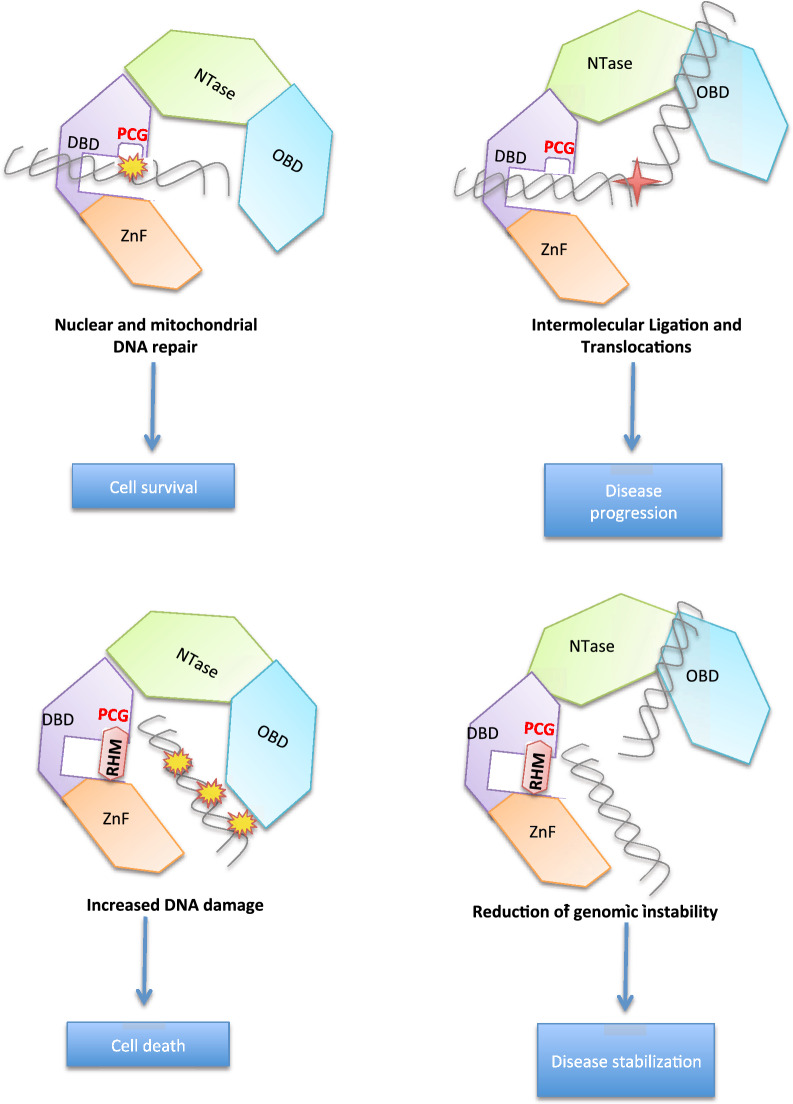
Schematic representation of proposed RHM mechanism of action. RHM binds to the PCG on LIG3 DBD, impairing ZnF-DBD interaction. This event reduces DSB recognition, repair and chromosome translocations, thus inducing MM cell death and disease stabilization

Although further investigation is required to finally validate the specific binding site on LIG3 and dissect the mechanisms of anti-tumor activity, and integrative multi-omics platforms at different molecular levels might provide valuable insights into the complexity of this biological system [71–77], based on our findings, we could hypothesize that RHM impairs DNA Ligase III activity by binding to PCG of DBD, thus hampering DNA damage recognition, repair and chromosome translocations (Fig. 6).

## Conclusions

Overall, our data demonstrate that RHM has powerful anti-MM activity by targeting LIG3-driven DNA repair and confirm that MM cells are Alt-NHEJ-MYC-addicted. Even if a variety of natural compounds have shown antitumor activity in preclinical models of MM [78, 79], in this study, we provide the first evidence of anti-MM activity induced by RHM, selected after an unbiased multistep screen path, and functionally validated.

Importantly, our findings provide a framework for a potential novel agent in Alt-NHEJ-dependent HR-deficient tumors, with a safer toxicity profile as compared to PARP-inhibition. We provide a rationale for future studies of RHM as a diet supplement to reduce the risk of progression to overt symptomatic disease of MGUS or SMM patients, or in relapsed/refractory MM patients, as a safe and sustainable chemo-free investigational strategy [80].

## Supplementary Information

**Additional file 1: Supplementary Figures Figure S1.** A Cartoon backbone representation of the superimposed DNA Binding Domains (DBD) of DNA Ligase I (green), III (orange) and IV (blue). B. Surface view of the DBD of ligase III (PDB ID 3L2P) . The positively charged groove targeted in the virtual screening is encircled by the dashed line. C. Surface view of the DBD of ligase I (PDB ID 1X9N). D. Surface view of the DBD of ligase IV (PDB ID 3W5O). **Figure S2.** **A** Immunoblot analysis of LIG4 expression in MM cell lines. GAPDH was used as loading control. **B** Representative FACS traces of Annexin V assay in AMO1 and H929 cells 4 days after RHM treatment (5 μM). **C** H929 cells were treated with vehicle or RHM (5 μM). 6 hours after either DMSO (NT) or Z-VAD-FMK were added to culture medium, at final concentration of 25 μM. Annexin V staining 4 days after treatment is shown. **D** Annexin V assay on U266 cells after 4 days of treatment with vehicle or RHM (5 μM). **E** Colony formation of AMO1 cells treated with vehicle or increasing dose of RHM. Light microscopy after 2 weeks is shown. **F** H&E stain of AMO1 and KMS26 cells after 5 days of treatment with vehicle or RHM (5 μM). **Figure S3.** **A** Cell cycle analysis in H929 cells treated with RHM (5 μM) with or without Caffeine (5 mM) for 48h. **B** ROS-Glo H2O2 assay performed in AMO1 and KMS26 cells 48h after treatment with vehicle or RHM (5 μM). **C** Relative percentage of CNV acquisition in AMO1 treated with RHM (2,5 μM) or vehicle, as evaluated by Affymetrix CytoScan HD Array analysis. **Figure 4.** **A** Cell viability of AMO1 and ABZB, treated with vehicle or increasing dose of Bortezomib for 48h. **B** Graphs of correlations between endogenous mRNA expression levels of LIG3 and MYC in MM patients with EFS<24 months, from GSE24080 public dataset. **C** Immunoblot analysis of MYC expression in AMO1 and ABZB cells. GAPDH was used as loading control.

**Additional file 2: Supplementary Methods **

## Data Availability

MAQC-II Project: MM data set, Series GSE24080.
