# Supplementary material for: Exploiting DNA Ligase III addiction of multiple myeloma by flavonoid Rhamnetin
Source: J Transl Med. 2022 Oct 22;20:482. doi: 10.1186/s12967-022-03705-z (PMC9588242; doi:10.1186/s12967-022-03705-z)
Supplement: Supplementary file 1 — Additional file 1: Supplementary Figures Figure S1. A Cartoon backbone representation of the superimposed DNA Binding Domains (DBD) of DNA Ligase I (green), III (orange) and IV (blue). B. Surface view of the DBD of ligase III (PDB ID 3L2P) . The positively charged groove targeted in the virtual screening is encircled by the dashed line. C. Surface view of the DBD of ligase I (PDB ID 1X9N). D. Surface view of the DBD of ligase IV (PDB ID 3W5O). Figure S2. A Immunoblot analysis of LIG4 expression in MM cell lines. GAPDH was used as loading control. B Representative FACS traces of Annexin V assay in AMO1 and H929 cells 4 days after RHM treatment (5 μM). C H929 cells were treated with vehicle or RHM (5 μM). 6 hours after either DMSO (NT) or Z-VAD-FMK were added to culture medium, at final concentration of 25 μM. Annexin V staining 4 days after treatment is shown. D Annexin V assay on U266 cells after 4 days of treatment with vehicle or RHM (5 μM). E Colony formation of AMO1 cells treated with vehicle or increasing dose of RHM. Light microscopy after 2 weeks is shown. F H&E stain of AMO1 and KMS26 cells after 5 days of treatment with vehicle or RHM (5 μM). Figure S3. A Cell cycle analysis in H929 cells treated with RHM (5 μM) with or without Caffeine (5 mM) for 48h. B ROS-Glo H2O2 assay performed in AMO1 and KMS26 cells 48h after treatment with vehicle or RHM (5 μM). C Relative percentage of CNV acquisition in AMO1 treated with RHM (2,5 μM) or vehicle, as evaluated by Affymetrix CytoScan HD Array analysis. Figure 4. A Cell viability of AMO1 and ABZB, treated with vehicle or increasing dose of Bortezomib for 48h. B Graphs of correlations between endogenous mRNA expression levels of LIG3 and MYC in MM patients with EFS<24 months, from GSE24080 public dataset. C Immunoblot analysis of MYC expression in AMO1 and ABZB cells. GAPDH was used as loading control. [file 12967_2022_3705_MOESM1_ESM.pdf]

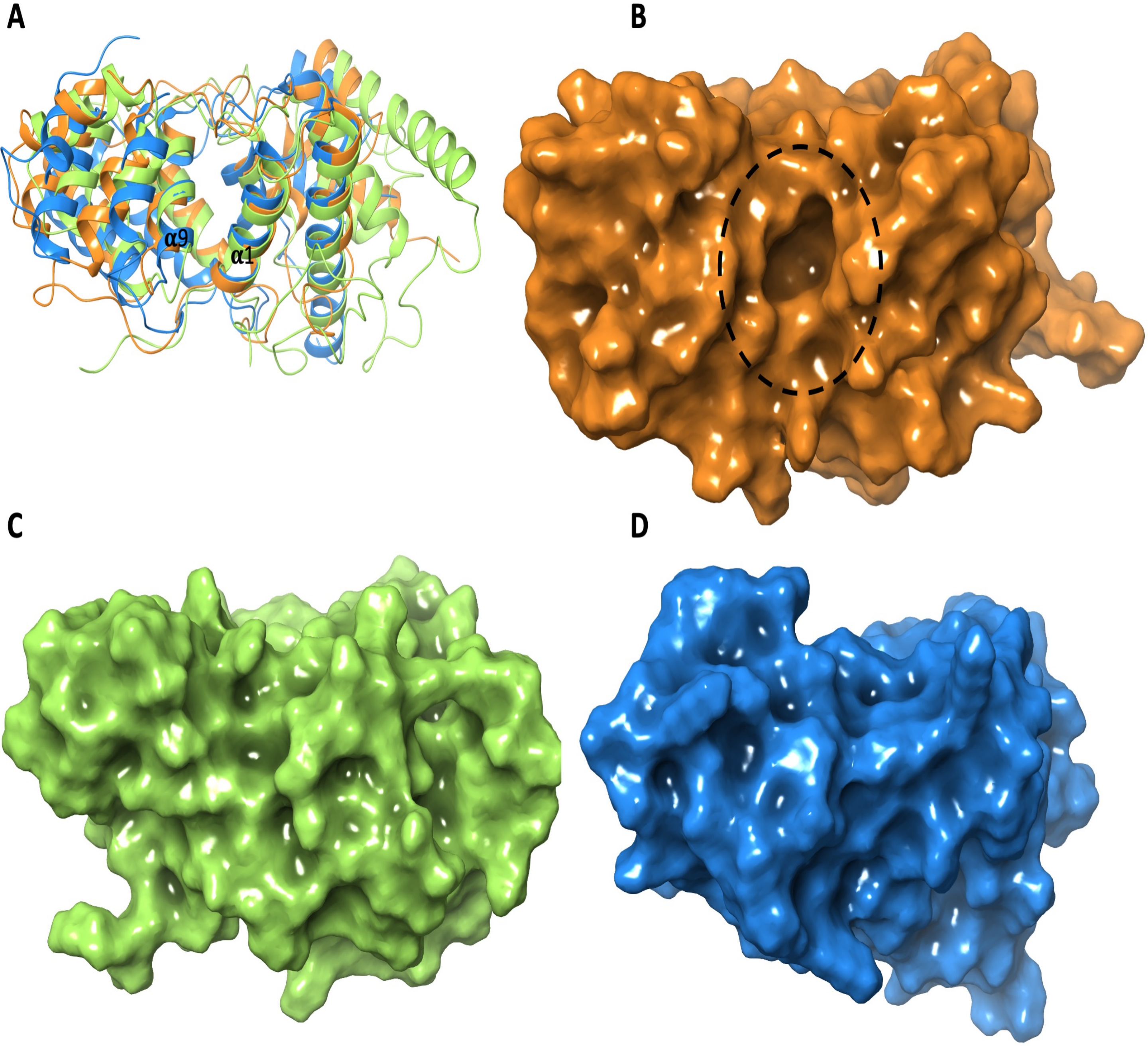

Supplementary Figure 1. A. Cartoon backbone representation of the superimposed DNA Binding Domains (DBD) of DNA Ligase I (green), III (orange) and IV (blue). B. Surface view of the DBD of ligase III (PDB ID 3L2P) . The positively charged groove targeted in the virtual screening is encircled by the dashed line. C. Surface view of the DBD of ligase I (PDB ID 1X9N). D. Surface view of the DBD of ligase IV (PDB ID 3W5O).

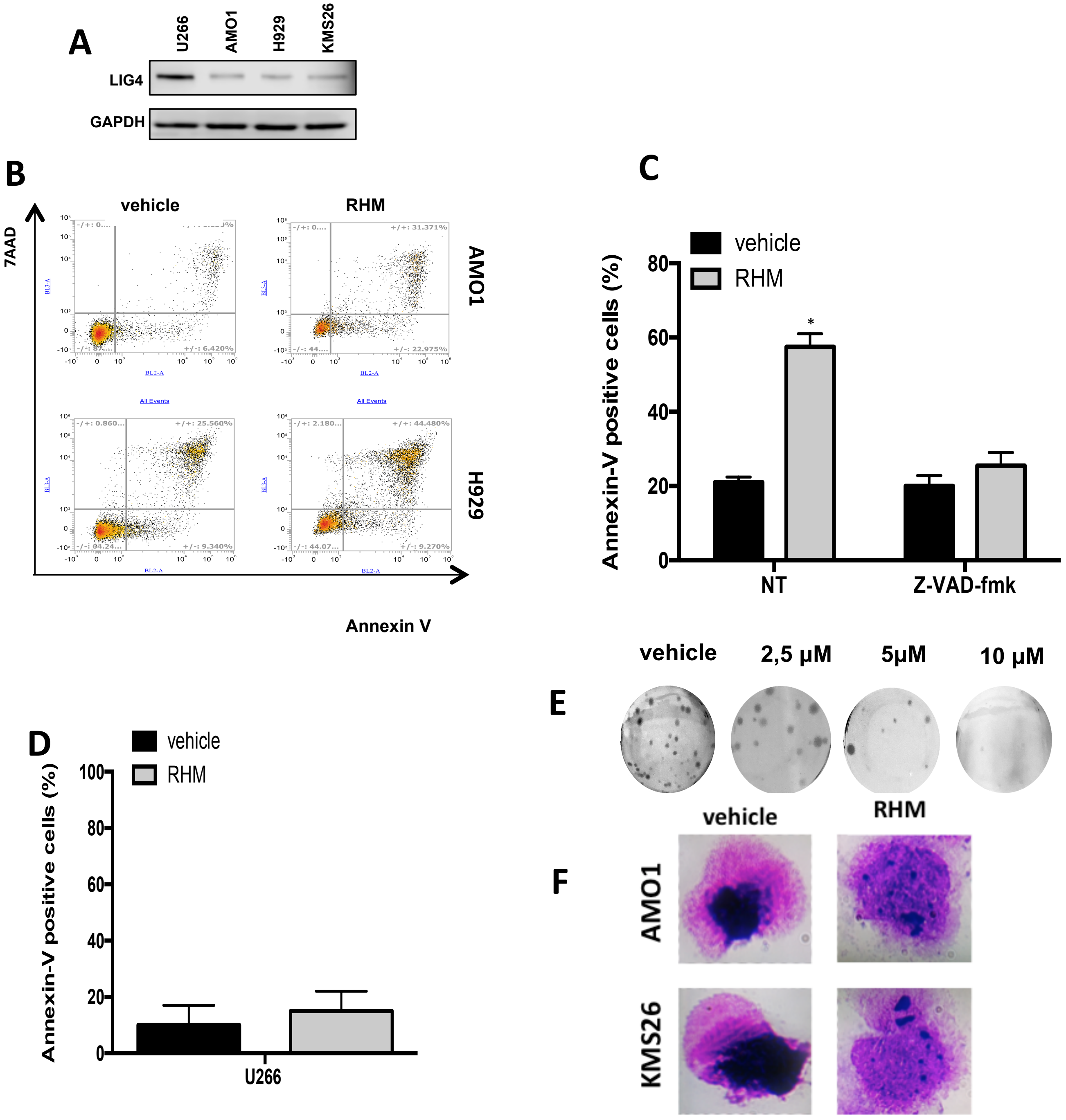

Supplementary Figure 2. A. Immunoblot analysis of LIG4 expression in MM cell lines. GAPDH was used as loading control. **B.** Representative FACS traces of Annexin V assay in AMO1 and H929 cells 4 days after RHM treatment (5 µM). **C.** H929 cells were treated with vehicle or RHM (5 µM). 6 hours after either DMSO (NT) or Z-VAD-FMK were added to culture medium, at final concentration of 25 µM. Annexin V staining 4 days after treatment is shown. **D.** Annexin V assay on U266 cells after 4 days of treatment with vehicle or RHM (5 µM). **E.** Colony formation of AMO1 cells treated with vehicle or increasing dose of RHM. Light microscopy after 2 weeks is shown. **F.**H&E stain of AMO1 and KMS26 cells after 5 days of treatment with vehicle or RHM (5 µM).

.

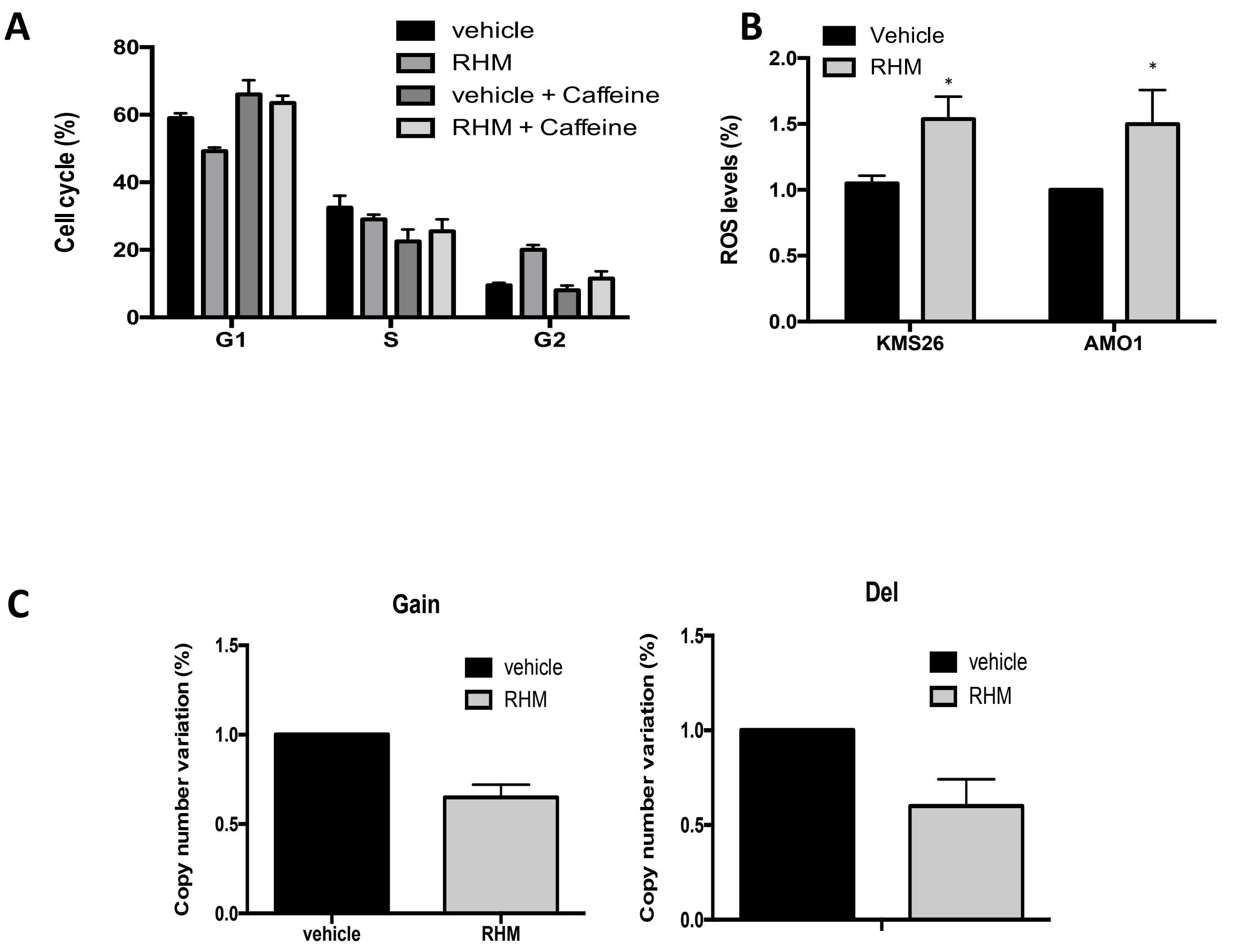

Supplementary Figure 3. A. Cell cycle analysis in H929 cells treated with RHM (5 µM) with or without Caffeine (5 mM) for 48h. **B.** ROS-Glo H2O2 assay performed in AMO1 and KMS26 cells 48h after treatment with vehicle or RHM (5 µM). **C.** Relative percentage of CNV acquisition in AMO1 treated with RHM (2,5 µM) or vehicle, as evaluated by Affymetrix CytoScan HD Array analysis.

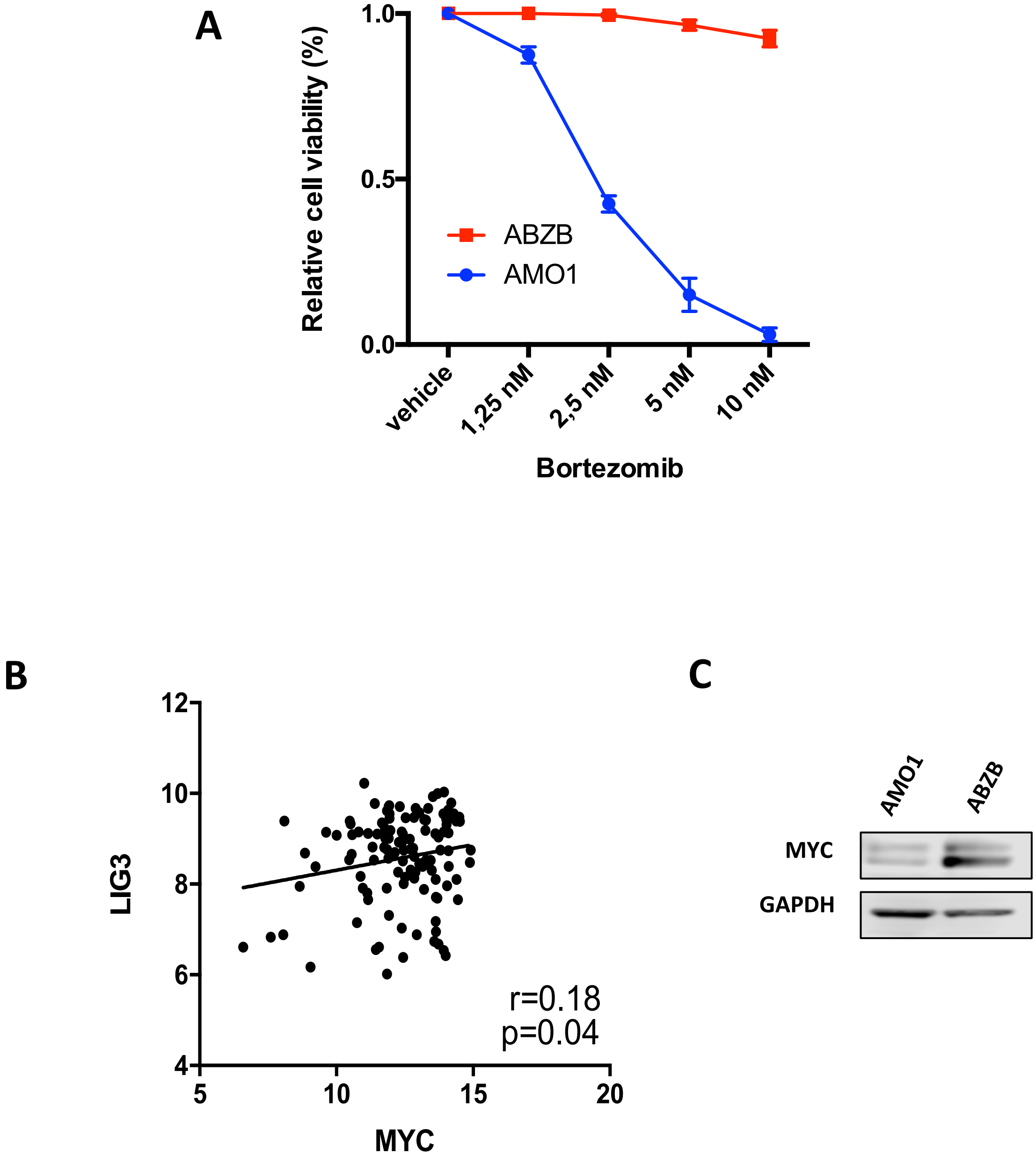

Supplementary Figure 4. A. Cell viability of AMO1 and ABZB, treated with vehicle or increasing dose of Bortezomib for 48h. **B.** Graphs of correlations between endogenous mRNA expression levels of LIG3 and MYC in MM patents with EFS<24 months, from GSE24080 public dataset. **C.** Immunoblot analysis of MYC expression in AMO1 and ABZB cells. GAPDH was used as loading control.
