# Supplementary material for: Exploiting DNA Ligase III addiction of multiple myeloma by flavonoid Rhamnetin
Source: J Transl Med. 2022 Oct 22;20:482. doi: 10.1186/s12967-022-03705-z (PMC9588242; doi:10.1186/s12967-022-03705-z)
Supplement: Supplementary file 2 — Additional file 2: Supplementary Methods [file 12967_2022_3705_MOESM2_ESM.pdf]

Supplementary Methods

Nuclear Magnetic Resonance (NMR) experiments

Recombinant Human LIG3 protein was purchased by Creative BioMart (Shirley, NY, USA) and it was stored at a 50µM concentration in phosphate buffer. Rhamnetin (RHM) ($C_{16}H_{12}O_7$), was purchased by Merck Life Science S.r.l..

NMR experiments were performed on a 600 MHz Bruker Avance spectrometer at 298 K in deuterated phosphate buffer 20 mM pH=7.4 with 150 mM NaCl. RHM was characterized through 1D 1H spectra, 2D 1H-1H COSY, TOCSY, NOESY and HSQC experiments. RHM samples were prepared adding a percentage lower than 10% of DMSO-d6.

STD NMR experiments were performed using WATERGATE 3-9-19 pulse sequence for water suppression. On-resonance irradiation of the protein was performed at a chemical shift of -0.05 ppm; off-resonance irradiation was applied at 200 ppm, where no protein signals are visible. Selective presaturation of the protein was achieved by a train of Gauss-shaped pulses of 49 ms length each. The STD spectra were acquired with varying saturation times from 0.98 s to 2.94 s; the optimized total length of the saturation train was 2.94 s for all compounds. Blank experiments were conducted in absence of protein in order to avoid artefacts. Intensities of all STD effects (absolute STD) were calculated by division through integrals over the respective signals in STD reference spectra.

$\frac{I_{0-}I_{sat}}{I_0}$ is the fractional STD effect, expressing the signal intensity in the STD spectrum as a fraction of the intensity of an unsaturated reference spectrum. In this equation, I_0 is the intensity of one signal in the off-resonance or reference NMR spectrum, Isat is the intensity of a signal in the on-resonance NMR spectrum, and I_0 − Isat represents the intensity of the STD spectrum.

For STD NMR experiments, all protein/ligand samples were prepared in a 1:100 protein/ligand ratio. The final concentration of the samples was 10 µM of protein and 1 mM of ligand, and the final volume was 500 µL.

ChIP

1.5 $\times 10^7$ AMO1 and ABZB cells were crosslinked in 1% formaldehyde, lysed and sheared by sonication for 10 cycles (each of 30 seconds) by using the Bioruptor Plus (Diagenode). Chromatin was divided into equal amounts of immunoprecipitation with the MYC antibody (ab56), or rabbit IgG as negative control (Santa Cruz Biotechnology). Chromatin extracts were incubated on a rotator with 20 ml of ChIP Grade Protein A/G Plus Agarose for 3 h at 4°C. Then, bound agarose beads were

harvested by centrifugation (12.000 rpm, 15 seconds) and washed; the precipitated protein-DNA complexes were next eluted from washed beads and incubated twice at 65°C for 1.5 h with NaCl and Proteinase K to revert cross-links.

Purified DNA was then subjected to qPCR using GoTaq qPCR Master Mix (Promega). Primer sequences for qPCR were:

LIG3(forward) $^{5'}$-AACTACTCCCAAACATCACAGG-$^{3'}$

LIG3(reverse) $^{5'}$-CTTTAAATCCGGGTCCTAGAGC-$^{3'}$

Promoter activity assay

ABZB cells were co-transfected with siRNA control or MYC–targeting siRNA and negative control (NEGPG04) or LIG3 (HPRM44239-PG04) promoter constructs (GeneCopeia, Rockville, MD, USA). Measurement of promoter activity was performed with Secrete-Pair ™ Dual Luminescence Assay Kit (GeneCopeia, Rockville, MD, USA) according to manufacturers's instructions

SNP-array data analysis

DNA was extracted from the AMO1 treated for 1 month with vehicle or RHM (2,5 μM), using the Perfect Pure DNA Blood kit (5 Prime) and analyzed using the Affymetrix Cytoscan HD array (Affymetrix, Inc., Santa Clara, CA) according to manufacturers's instructions, to evaluate genomic instability and ongoing DNA rearrangements. This array consists of 2.67 million markers for copy number variation (CNV) analysis, including 750,000 SNP and 1.9 million non-polymorphic probes, with an average spacing for RefSeq genes of 880 bp. Analysis of intensity data (CEL file) was performed with Chromosome Analysis Suite v 3.1 (ChAS 3.1) software using the Affymetrix HapMap Reference Model File for comparison. We used 25 probes and > 25 kb and >50 kb as a minimum cutoff for deletions and gains respectively. Map position was based on GRCh37/hg19 assembly.

Mitochondrial morphology

Mitochondria staining were performed by incubation with 250 nmol/L Mitotracker Red FM (Thermo Scientific) for 20 minutes at 37 C. After washing, cells were harvested, centrifuged onto glass slides (Cytospin 4, Thermo Scientific). Images were acquired with an SP2 Leica Zeiss confocal laser-scanning microscope with a 63× oil objective.

Mitochondrial DNA measurements

To assess mitochondrial DNA (mtDNA) copy number, genomic DNA was extracted from cells using the Perfect Pure DNA Blood kit (5 Prime). The relative mtDNA copy

number was determined by a real-time polymerase chain reaction, according manufacturer's instructions (human real-time PCR mitochondrial DNA damage analysis kit, Detroit R&D).

Histology and Immunohistochemistry

Retrieved tumors from animals were fixed in 4% buffered formaldehyde and 24 hours later washed, dehydrated, and embedded in paraffin. For light microscopy analysis by an optical microscope Nikon i55 (Nikon Corporation, Tokyo, Japan), we performed staining with H&E on 4-mm tumor sections mounted on poly-lysine slides. For IHC staining, 2-mm–thick tumor slices were de-paraffinized and pretreated with the Epitope Retrieval Solution 2 (EDTA buffer, pH 8.8) at 98 C for 20 minutes. After washing steps, peroxidase blocking was carried out for 10 minutes using the Bond Polymer. All procedures were performed using the Bond Max Automated Immunohistochemistry. Tissues were washed and incubated with the primary antibody directed against Ki-67 (Dako, clone MIB-1; 1:150). Subsequently, tissues were incubated with polymer for 10 minutes and developed with DAB–Chromogen for 10 minutes. Slides were counterstained with Hematoxylin.

Western blot analysis

Whole cell protein extracts were prepared from MM cells and from PBMCs in NP40 CellLysis Buffer (Novex®) containing a cocktail of protease inhibitors (Sigma, Steinheim, Germany). For fractionated extraction protocol, Subcellular Protein Fractionation Kit for Cultured Cells (ThermoFisher) was used. Cell lysates were loaded and PAGE separated. Proteins were transferred by Trans-Blot® TurboTM Transfer Starter System for 7 min. After protein transfer, the membranes were blotted with antibodies listed in the table and visualized with C-DiGit® Blot Scanner (LI-COR) by using the ECL Western Blotting Detection Reagents (Thermo Fisher Scientific, IL). Image capture was carried out using image studio® (LI-COR, version 5.0) software.

**Antibodies**	**Sources**	**Catalog #**	**Applications**
Cleaved-Caspase 3 (Asp175)	Cell Signaling Technology	9661	WB (1:1000)
GAPDH	Santa Cruz	25778	WB (1:3000)

Cleaved PARP (Asp214)	Cell Signaling Technology	9544	WB (1:1000)
Phospho-CHK1 (Ser345)	Cell Signaling Technology	2348	WB (1:1000)
Phospho-CHK2 (Thr 68)	Cell Signaling Technology	2197	WB (1:1000)
phospho-Histone H2A.X (Ser139)	Cell Signaling Technology	9718	WB (1:1000)<br>IF (1:200)
c-Myc	Cell Signaling Technology	5605	WB (1:1000)
LIG3	Abcam	ab96576	WB (1:1000)
Goat anti-mouse IgG-HRP	Santa Cruz	2055	WB (1:3000)
Goat anti-rabbit IgG-HRP	Santa Cruz	2054	WB (1:3000)

List of Antibodies

WB, western blot. ChIP, chromatin immunoprecipitaion. IF, immunofluorescence.
